# Genetic Variation of *Schizothorax wangchiachii* Populations Between the Jinsha and Yalong Rivers Using Simplified Genome Sequencing

**DOI:** 10.3390/ani16050802

**Published:** 2026-03-04

**Authors:** Taiming Yan, Ping Chen, Qinyao Tian, Huiling Wang, Hongjun Chen, Ziting Tang, Zhen Wei, Yinlin Xiong, Deying Yang, Zhi He

**Affiliations:** 1 Fisheries College, Sichuan Agricultural University, 211# Huimin Road, Chengdu 611130, China; yantaiming@sicau.edu.cn (T.Y.);; 2 Center for Conservation and Utilization of Rare and Endemic Fishes in Sichuan, Chengdu 611230, China

**Keywords:** genetic diversity, population structure, genotyping-by-sequencing, *Schizothorax wangchiachii*

## Abstract

*Schizothorax wangchiachii*, as a cold-water fish, is a predominant or common species in high-altitude areas with large population sizes. It is among the main edible economic fish species in the production area. We used genotype sequencing (GBS) technology to analyze the genetic diversity and population structure of 10 wild populations in the Jinsha River and Yalong River basins of the upper Yangtze River. A total of 724,858 single-nucleotide polymorphisms (SNPs) were detected in these 10 wild populations. The population genetic diversity was low; however, the degree of genetic differentiation was not significant. The populations from the Jinsha River and Yalong River could not be clustered separately on the basis of the SNPs. The Panzhihua (PZH) and Wudongde (WDD) populations from the Jinsha River exhibited gene flow with the Yajiang (YJ) population from the Yalong River, and a secondary pulse of gene flow subsequently connected the PZH and WDD populations to the upper-Jinsha population Suwalong (SWL). The demographic history of *S. wangchiachii*, reflected in its effective population size (*N_e_*), has been influenced by the uplift of the Qinghai–Tibet Plateau (QTP). Furthermore, the identified SNPs are functionally associated with key cellular processes, environmental adaptation, and metabolism. These findings provide critical genomic insights that can inform conservation strategies and support the sustainable utilization of wild *S. wangchiachii* resources.

## Simple Summary

Analysis of SNPs obtained from GBS sequencing revealed that the 10 wild populations of *Schizothorax wangchiachii* from the upper Yangtze River exhibit low genetic diversity and a simple population structure. The populations from Jinsha River can be separated from those of the Yalong River. The upstream of Jinsha River (including BD, GT, and SWL populations) should be established as a nature reserve for *S. wangchiachi*. Energy metabolism and cellular processes may contribute to adaptation of high-altitude and low-temperature environments. The results of this study can provide a direct basis for formulating conservation strategies for *S. wangchiachii*.

## 1. Introduction

The Hengduan Mountains are located at the eastern edge of the Qinghai–Tibet Plateau and are recognized as biodiversity hotpots worldwide, and they contain abundant and unique fish species [1,2,3,4,5]. The clustering results for fish species other than plateau fish indicate that the similarity between water systems in the Hengduan Mountains changes from west to east. The isolation of various water systems in the region from west to east can be attributed to the uplift of the plateau. The complexity of the geological structure of the Hengduan Mountains notably complicates the hydrological evolution process in this area even more complex. The Hengduan Mountains have formed a clear natural barrier between the Yalong River and the Jinsha River, facilitating their separation [6]. *Schizothorax wangchiachii*, a fish endemic to the Jinsha River and Yalong River basins in this region, is not only a typical representative of plateau fish fauna but also an ideal model for studying the evolution and adaptation mechanism of fishes on the eastern margin of the Qinghai–Tibet Plateau (QTP) [7]. However, in recent years, the abundance of *S. wangchiachii* has declined dramatically due to overfishing, mining, hydropower development in tributaries, and broader environmental degradation [8,9]. Moreover, planned construction of dam cascades on the Jinsha River mainstream is projected to exacerbate this population decline [10,11,12]. Consequently, a comprehensive assessment of its current population status and genetic resources is urgently needed.

The complex topography and diverse ecological environment of the Jinsha River and Yalong River basins provide abundant habitats for *S. wangchiachii* and may drive population differentiation and adaptive evolution. Thus, a comprehensive understanding of the genetic diversity and population structure of these fish is essential for formulating effective conservation measures and breeding strategies. Previous studies have focused mainly on early embryonic development, growth and reproductive characteristics, and the behavioral ecology of *S. wangchiachii* [13,14,15]. Population genetic analysis is a fundamental tool for assessing population divergence and informing conservation strategies [16]. However, genetic information concerning molecular markers in *S. wangchiachii* is very limited. SLAF-seq (specific-locus amplified fragment sequencing) technology was used to develop SNP markers for *S. wangchiachii*, and a moderate genetic diversity level of the natural population of *S. wangchiachii* in the Ahai and Longkoujiang sections of the Jinsha River was revealed in 2018 [17]. In a 2008 study conducted in the middle reaches of the Yalong River, the genetic diversity of *S. wangchiachii* was assessed using random amplified polymorphic DNA (RAPD) markers, revealing a relatively low level of diversity [18]. However, research on the genetic differentiation patterns and driving mechanisms of the *S. wangchiachii* population in this watershed is insufficient, and a systematic genetic structure analysis is lacking. Therefore, a systematic study of the genetic structure of *S. wangchiachii* in these two watersheds is warranted. Such research is crucial to assess the impacts of human activities (e.g., hydropower development and overfishing) on its genetic diversity and to elucidate the potential evolutionary roles of historical factors, such as the uplift of the QTP and changes in river systems.

Genotyping by sequencing (GBS) is a simplified genome sequencing method capable of detecting numerous single-nucleotide polymorphisms (SNPs), thereby elucidating evolutionary relationships among species and population genetic structures [19,20,21]. This highly efficient GBS approach can uncover tens of thousands of genomic variants and deliver comprehensive genotypic data at the genome level [22,23]. Furthermore, SNPs derived from GBS are notably abundant, exhibit lower mutation rates, and can be genotyped with reduced error rates [24]. Most karyologically-studied Schizothoracinae species are polyploid (dominantly tetraploid; some hexaploid), with chromosome counts from ~66 to ~148 (and reports of higher counts); tetraploids with 2n ≈ 92 or 98 are common patterns [25,26,27,28]. Previous studies have demonstrated the utility of GBS in addressing various evolutionary biology questions in Schizothorax [20,22,29]. To date, the chromosome-level genome of S. wangchiachii has not been reported. Therefore, it is feasible to use GBS to analyze the genetic diversity and population structure of the wild *S. wangchiachii* populations. In our study, 10 wild populations of *S. wangchiachii* were collected from the main stems of the Jinsha River, Yalong River, and their tributary, the Xianshui River. Then, the genetic diversity, genetic differentiation, gene flow, demographic history, and the function enrichment of SNP were analyzed. These results provide important information for the protection, development and utilization of *S. wangchiachii*.

## 2. Materials and Methods

### 2.1. Sample Collection

**Figure 1:**
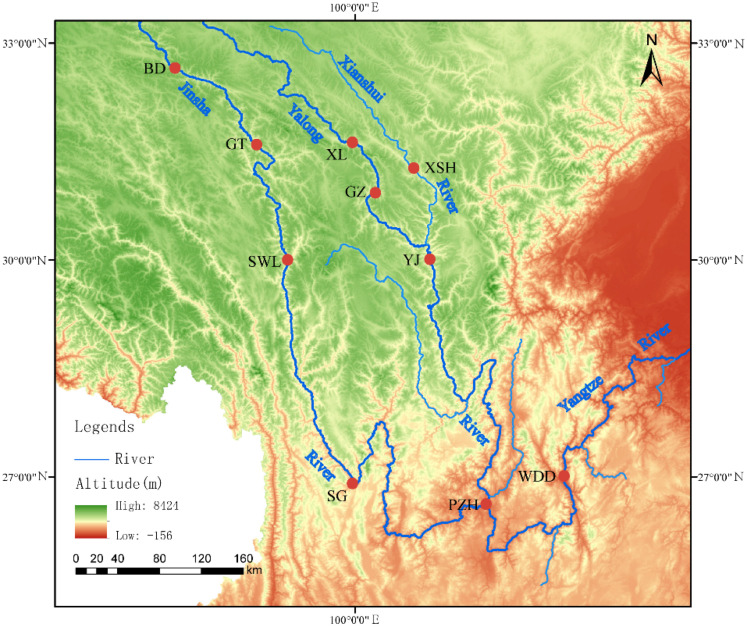
Map of the sampling sites. The map was drawn by ArcGIS10.8 software. BD, Benda; GT, Gangtuo; SWL, Suwalong; SG, Shigu; PZH, Panzhihua; XL, Xinlong; GZ, Ganzi; XSH, Xianshuihe; YJ, Yajiang; WDD, Wudongde.

Seventy-five samples of *S. wangchiachii* were collected from 10 sites in the Jinsha River [including Benda (BD), GangTuo (GT), Suwalong (SWL), Shigu (SG), Panzhihua (PZH), and Wudongde (WDD)], Yalong River [including Xinlong (XL), Ganzi (GZ), and Yajiang (YJ)] and its tributaries, namely, the Xianshui River (XSH). Sample information is shown in Figure 1 and detailed in Table 1. The dorsal fin of *S. wangchiachii* was clipped for the samples. All fin samples were preserved in 95% ethanol and maintained at −20 °C. All experimental protocols and investigations were reviewed and approved by the Animal Research and Ethics Committee of Sichuan Agricultural University (Approval No. 20220480) and were conducted in strict compliance with the committee’s established guidelines.

### 2.2. DNA Extraction and Sequencing and SNP Identification

Genomic DNA (gDNA) was isolated from frozen tissue using the TIANcombi DNA Lyse & Det PCR Kit (Tiangen Biotech, Beijing, China). The DNA quality was assessed by 1% agarose gel electrophoresis, and the concentration was measured with a NanoDrop 2000 spectrophotometer d (Thermo Scientific, Boston, MA, USA). All gDNA samples were standardized to an optimal concentration of 50 ng/µL for library construction. Sequencing libraries were prepared using a modified genotyping-by-sequencing (GBS) approach [30], performed by OE Biotech Co., Ltd. (Shanghai, China). Briefly, gDNA was digested with the restriction enzymes PstI-HF and MspI (NEB, Beverly, MA, USA). The resulting fragments were ligated to barcode adapters using T4 DNA ligase. Fragments ranging from 300 to 700 bp were selectively recovered using an optimized magnetic bead-based size selection system. The selected fragments were then amplified by PCR with a high-fidelity DNA polymerase. PCR product concentration was quantified using a Qubit fluorometer (Thermo Scientific), and only samples with concentrations exceeding 5 ng/µL were retained for sequencing. Final libraries were sequenced on an Illumina NovaSeq platform (Illumina, San Diego, CA, USA) with paired-end 150 bp reads.

Raw sequencing reads were processed to remove adapter sequences using the STACKS pipeline [31]. Quality filtering and the removal of low-quality reads were performed with FASTP [32] to generate clean reads. The cleaned reads were aligned to the reference genome of *Oxygymnocypris stewartia* [33] (GenBank accession: PRJNA557578) using BWA. The single-nucleotide polymorphism (SNP, only high-quality biallelic SNPs) and insertion/deletion (InDel) were identified by the haplotypecaller module in Gatk 4.1.3.0 software [34,35]. VCFtools 0.1.16 [35] was used to further filter as follows: first, the sites with a support depth of no less than 4 were retained; second, the loci with a minimum allele frequency (MAF) lower than 0.01 were deleted; third, at least 80% of the samples could be successfully typed. The high-quality SNPs were subsequently used for downstream population genomic analyses.

### 2.3. Genetic Diversity and Population Structure Analysis

Genetic diversity parameters, including the expected heterozygosity (*H_e_*), observed heterozygosity (*H_o_*), polymorphism information content (*PIC*), and nucleotide diversity (π) were assessed using VCFtools 0.1.16 [35]. The fixation index (*F_st_*) between populations was estimated with ARLEQUIN 3.5.1.3 [36].

The population genetic structure was inferred using ADMIXTURE v1.3.0 [37]. The optimal number of genetic clusters (K) was determined by selecting the value associated with the minimum cross-validation (CV) error, with K ranging from 1 to 7. Furthermore, the historical gene flow and population divergence among the three tributaries (Jinsha, Yalong, and Xianshui Rivers) were analyzed using Treemix v1.13 [38].

### 2.4. Phylogenetic Tree and Historic Population Dynamics Analysis

Phylogenetic relationships and population differentiation were assessed by constructing a neighbor-joining (NJ) tree [39]. The analysis utilized TreeBest 1.9.2 [40] to generate the genetic distance matrix, with branch support evaluated through bootstrapping (1000 replicates) [41]. Following phylogenetic reconstruction, the resulting tree was visualized using FigTree 1.4.4 software. Principal component analysis (PCA) based on the obtained SNP markers was performed using Plink2 1.90p software [42], with the first two principal components (PC1 and PC2) extracted for downstream analysis. Additionally, the effective population size (*N_e_*) trajectories of different geographical populations of *S. wangchiachii* were inferred using the SMC++ v0.6.5 software. The generation time (*g*) was set to 6 years, and the per-generation mutation rate (μ) was set to 4 × 10^−9^.

### 2.5. Gene Flow

The gene flow was analyzed by TreeMix v1.13 [38]. The analysis employed genome-wide allele frequency data to infer patterns of differentiation and admixture among multiple populations. This software took allele frequency data from multiple populations as input, generated maximum likelihood trees for these populations, and could also infer population admixture events. Prior to analysis, single-nucleotide polymorphisms (SNPs) were filtered with PLINK v1.90 [42] to minimize the effects of linkage disequilibrium. Migration events (m) ranging from 1 to 10 were tested, with five independent runs performed for each value of m. The likelihood values were −12,387.7 (m = 1), −11,377.8 (m = 2), −10,427.4 (m = 3), −10,079.5 (m = 4), and −9972.9 (m = 5). The optimal number of migration edges was determined using the R package OptM v0.1.6 (R v4.3.1). The resulting population graph was visualized with the plotting_funcs. R script was provided in the TreeMix 1.13 package.

## 3. Results

### 3.1. Sequencing Quality

A total of 69.76 Gb of raw sequencing data were generated from 75 samples. After quality control, 67.58 Gb of clean data were retained, yielding an average of 0.90 Gb per sample. The average sequencing depth across all samples was 7.06×. Using the previously published reference genome of *S. wangchiachii* [35], reads were aligned with a mapping rate ranging from 93.01% to 94.2%. The high sequencing quality was further confirmed by Q20 and Q30 scores, which exceeded 97.10% and 92.02%, respectively.

### 3.2. Clustering Analysis of Differential SNPs

**Figure 2:**
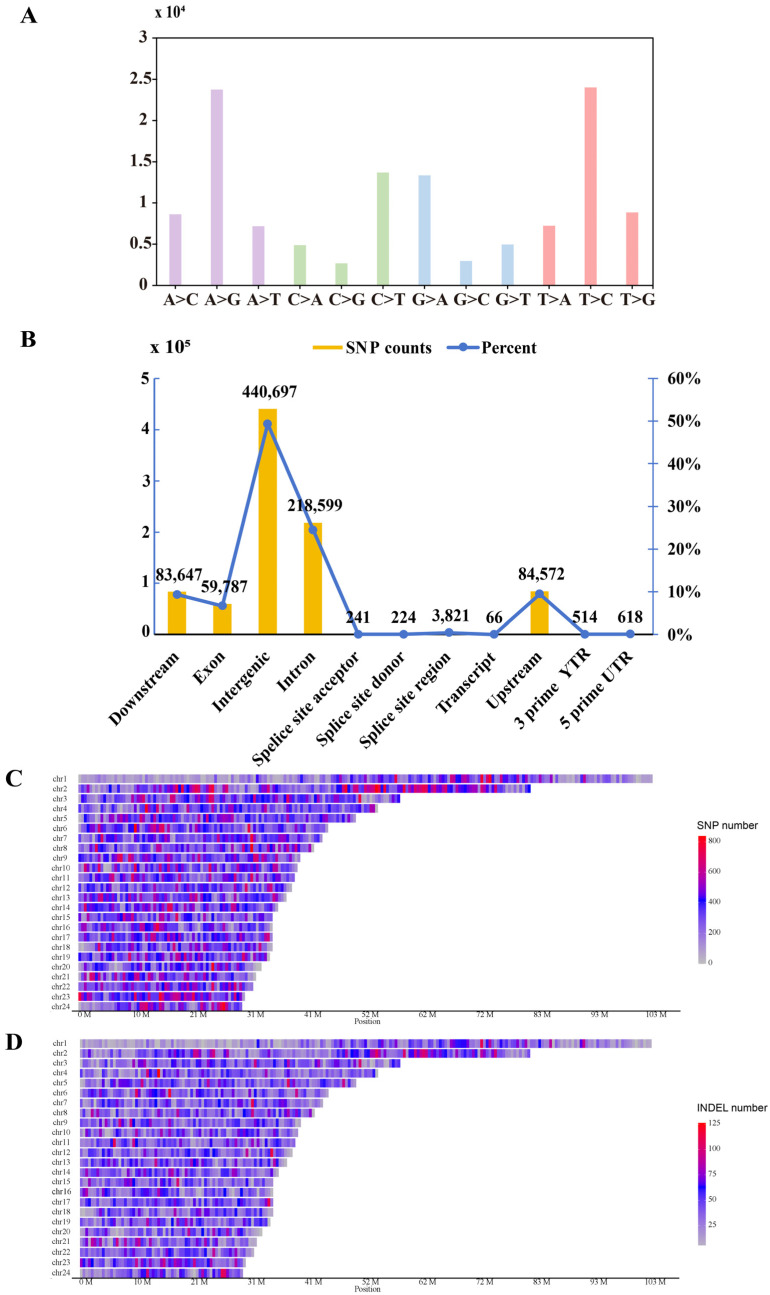
SNP and InDel information. (**A**), SNP type distribution. (**B**), Number of SNPs and InDels in different regions of the genome. (**C**,**D**), Density distribution of SNPs and InDels within 1 Mb on the chromosome, respectively. A, adenine deoxynucleotides; G, guanine deoxynucleotides; (**C**), cytosine deoxynucleotides; T, thymine deoxynucleotides; SNPs, single nucleotide polymorphisms; InDels, insertions and deletions; Chr1-24, chromosomes 1-24; Mb, megabase.

A total of 724,858 high-quality SNPs were identified through variant calling and stringent filtering. The detected nucleotide substitutions comprised both transitions and transversions, with transitions (A↔G/C↔T) being the predominant type (Figure 2A). The ratio of base transversions (total 4,274,331) to transitions (total 2,457,106) was 1.739579. Genomic annotation revealed that the majority of SNPs were located in intergenic and intronic regions, with additional variants distributed across exonic, upstream, and downstream regions (Figure 2B). Both SNPs and InDels showed notably high density on chromosomes 2 and 23 (Figure 2C,D), implying that these genomic regions may hold functional or evolutionary significance.

### 3.3. Population Genetic Diversity Analysis

The values of *H_e_*, *H_o_*, *P_i_*, and *PIC* in the SNP data ranged from 0.2200 to 0.2365, 0.2632 to 0.3411, 0.1058 to 0.2549 and 0.1738 to 0.1894, respectively, and all the populations exhibited moderate genetic diversity (Table 2). The observed heterozygosity was significantly higher than the expected heterozygosity, suggesting that the populations experienced nonrandom mating or recent population expansion. Notably, the nucleotide diversity displayed a clear geographic pattern: populations from the upper Jinsha River (BD, GT, SWL) were substantially higher than those from the lower basin. Although the content of polymorphic information was moderately polymorphic overall, its values in the upper-basin populations were significantly lower than those in the lower-basin populations.

Pairwise *F_st_* values and genetic distances among populations ranged between 0.0002 and 0.3963 and 0.0002 and 0.5047, respectively (Table 3). Population differentiation was assessed based on Wright’s *F_st_*, which served as the criterion for classification: values of 0 < *F_st_* ≤ 0.05 indicate low differentiation; 0.05 < *F_st_* ≤ 0.15, moderate differentiation; 0.15 < *F_st_* ≤ 0.25, high differentiation; and *F_st_* > 0.25, very high differentiation [43]. Analysis of pairwise *F_st_* values revealed that most population pairs of *S. wangchiachii* showed values less than 0.25, indicating an absence of pronounced genetic differentiation. The minimum *F_st_* was between populations BD and GT. The highest levels of genetic differentiation were detected in the comparisons of SG with GZ (*F_st_* = 0.3963) and SG with XL (*F_st_* = 0.3961). Additionally, the GZ-XL comparison yielded an *F_st_* of 0.3638, confirming a substantial degree of genetic divergence among these groups.

### 3.4. Population Structure and Systemic Development

**Figure 3:**
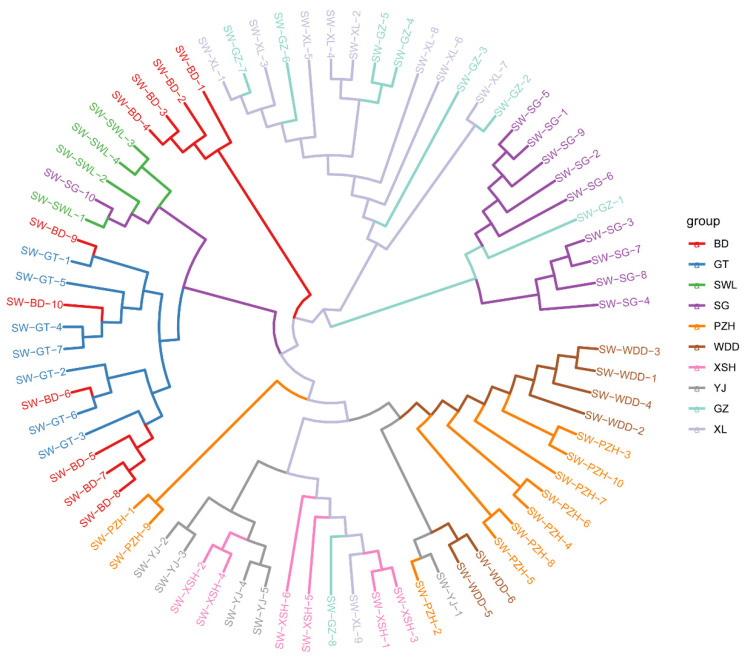
NJ phylogenetic tree of *Schizothorax wangchiachii* (SW) populations. BD, Benda; GT, Gangtuo; SWL, Suwalong; SG, Shigu; PZH, Panzhihua; XL, Xinlong; GZ, Ganzi; XSH, Xianshuihe; YJ, Yajiang; WDD, Wudongde. The Jinsha River includes BD, GT, SWL, SG, PZH and WDD. The Yalong River contains XL, GZ, YJ, and XSH.

The results of the NJ phylogenetic tree analysis showed that the 10 populations were intermixed and did not form distinct monophyletic clusters according to their population origins (Figure 3). Notably, a distinct phylogeographic pattern was observed, with clear lineage differentiation between the upstream populations of the Jinsha River (BD, GT, SWL, and SG) and both the downstream populations (PZH and WDD) and those from the Yalong River (XSH, YJ, GZ, and XL). Specifically, the YJ and XSH populations from the Yalong River water system formed a highly supported monophyletic clade with the PZH and WDD populations from the lower Jinsha River. However, genetic analysis revealed branch overlap and hybridization signals among select individuals from the GZ and XL populations of the middle reaches of the Yalong River, suggesting varying degrees of genetic introgression between different subgroups. This pattern likely reflects the ongoing gene flow resulting from frequent connectivity among *S. wangchiachii* populations.

**Figure 4:**
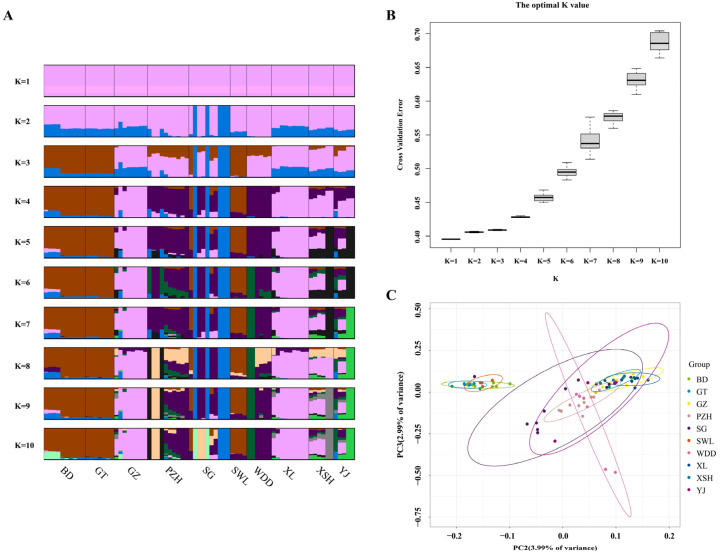
Principal component analysis and the error rate of the *Schizothorax wangchiachii* admixture K value. (**A**), The error rate of the *S. wangchiachii* admixture K value by cross-validation. (**B**), Clustering results of *S. wangchiachii*. (**C**), Principal component analysis for *S. wangchiachii*. BD, Benda; GT, Gangtuo; SWL, Suwalong; SG, Shigu; PZH, Panzhihua; XL, Xinlong; GZ, Ganzi; XSH, Xianshuihe; YJ, Yajiang; WDD, Wudongde.

The genetic clustering analysis across different assumed values of K revealed no clear genetic substructure corresponding to the geographic origins (i.e., river basins) of the sampling sites. All 75 individuals were grouped into a single genetic cluster only when the number of ancestral populations was set to one (K = 1; Figure 4A). This was further supported by the cross-validation error analysis, which identified K = 1 as the optimal value (Figure 4B). Consequently, based on the population genetic structure analysis, these 75 samples are considered to belong to a single panmictic population.

### 3.5. PCA

Based on 724,858 high-quality SNPs, the PCA analysis results revealed limited population structure. The first two principal components (PC1 and PC2) explained only 3.31% and 3.10% of the total genetic variance, respectively (Figure 4C). This low explanatory power indicates that the genetic variation captured by these SNPs was insufficient to differentiate the ten wild populations or to cluster the 75 individuals into distinct genetic subgroups.

### 3.6. Gene Flow Analysis

**Figure 5:**
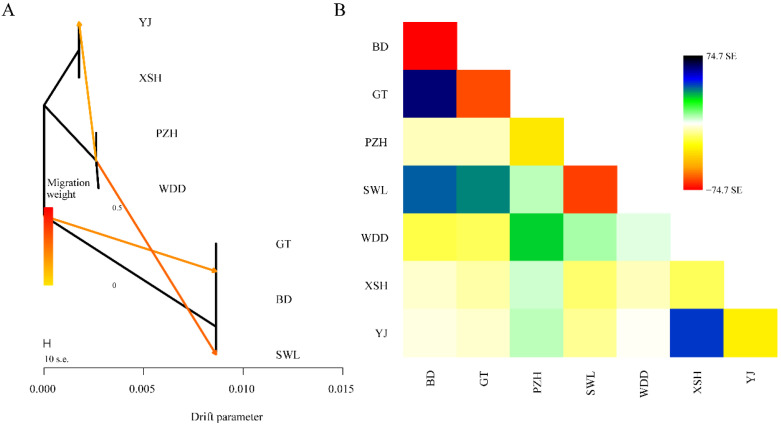
Gene flow evolutionary tree and heatmap of *Schizothorax wangchiachii.* (**A**), The gene flow evolutionary tree; the intensity of the red color in the arrows corresponds to the magnitude of gene flow, while the direction of the arrows indicates the direction of gene flow. (**B**), Heatmap of gene flow. BD, Benda; GT, Gangtuo; SWL, Suwalong; SG, Shigu; PZH, Panzhihua; XL, Xinlong; GZ, Ganzi; XSH, Xianshuihe; YJ, Yajiang; WDD, Wudongde. The Jinsha River includes BD, GT, SWL, SG, PZH and WDD. The Yalong River contains XL, GZ, YJ, and XSH. SE, standard error.

The gene flow ML evolutionary tree shows that the YJ and XSH populations in the Yalong River Basin and then the PZH and WDD populations of the lower reaches of Jinsha River began to differentiate, whereas the upper reaches of the Jinsha River began to differentiate. The PZH and WDD populations exhibited gene flow with the YJ population from the Yalong River, and a secondary pulse of gene flow subsequently connected PZH and WDD to the upper-Jinsha population SWL (Figure 5A,B). These sequential events imply that headwater isolation was followed by downstream expansion, with the lower Jinsha corridor serving as a recurrent conduit for bidirectional dispersal.

### 3.7. Historic Population Dynamics Analysis

**Figure 6:**
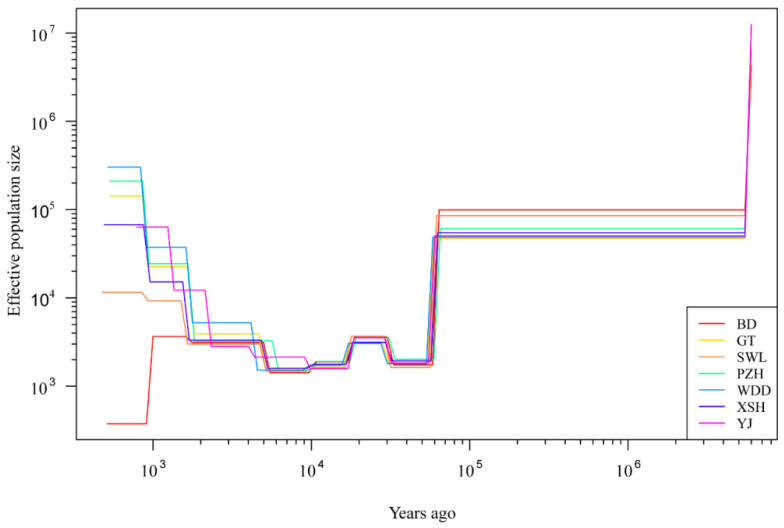
Demographic history of *Schizothorax wangchiachii.* BD, Benda; GT, Gangtuo; SWL, Suwalong; PZH, Panzhihua; XSH, Xianshuihe; YJ, Yajiang; WDD, Wudongde. The Jinsha River includes BD, SWL, PZH and WDD. The Yalong River contains YJ and XSH. Lines represent the median estimated *N_e_* of the seven groups.

All 10 populations exhibited a convergent demographic trajectory, characterized by a substantial expansion in effective population size (*N_e_*) from approximately 10 to 2.5 million years ago (Ma), spanning the Middle Miocene to the Pleistocene (Figure 6). This common trend likely reflects a period of ancient range expansion or population growth. Specifically, while the overall *N_e_* trends were largely congruent, the WDD and PZH populations shared a highly similar history, as did the XSH and YJ populations. Following this expansion, *N_e_* stabilized from about 1 Ma until 1 thousand years ago (Ka), after which all populations underwent a rapid decline.

### 3.8. Functional Annotations of the Selected SNPs

**Figure 7:**
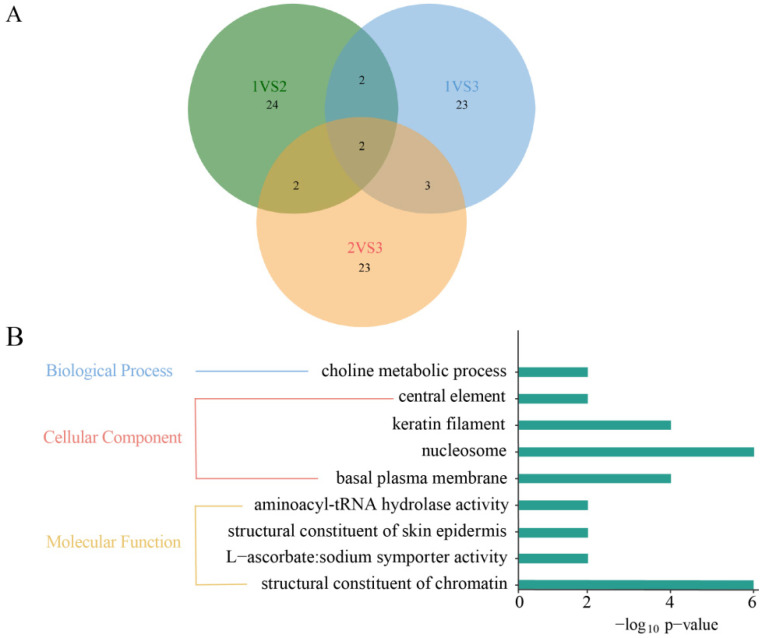
Functional annotation of selected SNPs based on KEGG pathway and GO terminology. (**A**), Venn diagram of significant KEGG pathways. (**B**), Nine common signaling pathways. Group 1: BD, GT, SWL and SG; Group 2: PZH and WDD; Group 3: XSH, YJ, GZ and XL. BD, Benda; GT, Gangtuo; SWL, Suwalong; PZH, Panzhihua; XSH, Xianshuihe; YJ, Yajiang; WDD, Wudongde.

On the basis of the results of the phylogenetic tree, PCA, and geographic information, the 10 populations of *S. wangchiachii* were merged into three groups (Group 1, BD, GT, SWL and SG; Group 2, PZH, WDD; Group 3, XSH, YJ, GZ and XL). The functional annotations of selected SNPs were based on the Kyoto Encyclopedia of Genes and Genomes (KEGG) and Gene Ontology (GO) enrichments. KEGG pathways and GO terms that were significantly enriched (*p* ≤ 0.05) for the differentially selected SNPs between the two groups were identified. (Figure 7). The top 30 GO enrichment analysis terms for each group (GO terms whose corresponding number of DEGs was higher than two in the three categories were then sorted in descending order of their −log10 *p* value for each item), and two common terms (nucleosome structural constituents of chromatin and cellular response to fructose stimulus) were screened. Among them, Group 1 vs. Group 2 and Group 1 vs. Group 3 (basal plasma membrane, L-ascorbic acid transmembrane transport activity) and Group 2 vs. Group 3 (keratin film, structural constant of skin epidermis) were separately determined for two common terms, whereas Group 1 vs. Group 3 and Group 2 vs. Group 3 were screened for three common terms (choke metabolic process central element and aminoacyl-tRNA hydrolase activity).

**Figure 8:**
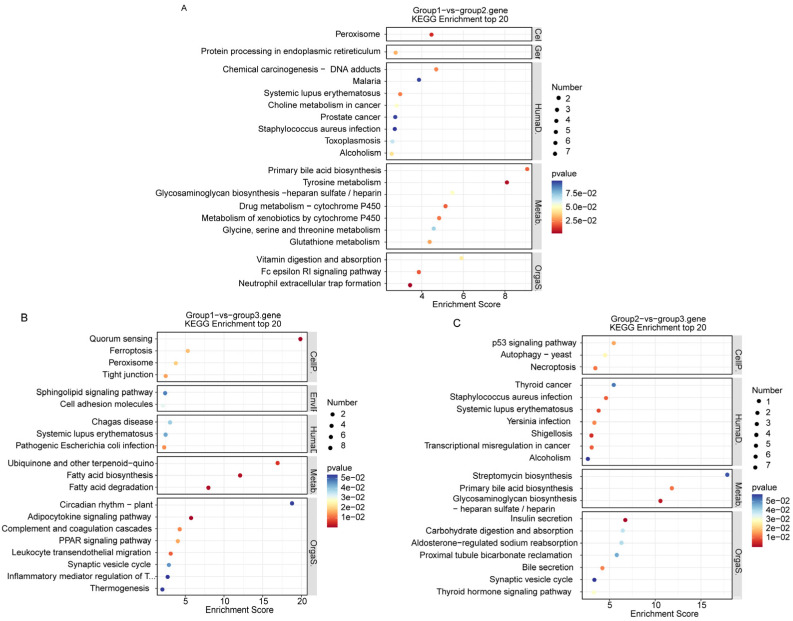
KEGG Enrichment Top 20 pathways. (**A**) Group 1 vs. group 2; (**B**) group 1 vs. group 3; (**C**) group 2 vs. group 3. Group 1: BD, GT, SWL and SG; Group 2: PZH and WDD; Group 3: XSH, YJ, GZ and XL. BD, Benda; GT, Gangtuo; SWL, Suwalong; PZH, Panzhihua; XSH, Xianshuihe; YJ, Yajiang; WDD, Wudongde.

Pathways significantly enriched in genes encoding differentially expressed proteins were identified on the basis of the KEGG database. On the basis of the top 20 pathway entries with a corresponding number of differentially expressed genes greater than two and a log10 *p* value for each entry, the three groups were collectively screened for one important KEGG pathway, namely, systemic lupus erythematosus, which is related to human activities (Figure 8). In addition, a common pathway for peroxisome and primary bile acid biosynthesis was found in Group 1 vs. Group 2 and Group 1 vs. 3 and Group 2 vs. 3, respectively.

## 4. Discussion

### 4.1. Basic Characteristics of the Genome and Levels of Genetic Diversity

Reduced representation sequencing enables accessing the fundamental characteristics of the genomes of species that lack prior genomic resources [44,45]. In this study, many genome fragments were obtained for 10 populations of *S. wangchiachii* by GBS. Analysis of the DNA base composition revealed that the percentage of GC content in *S. wangchiachii* reached 44.4%, which is higher than that in *G. przewalskii* (38.34%) [46] but lower than that in *S. kozlovi* (45%) [29]. The genomic GC content is not only correlated with genome size but also plays a critical role in shaping genome function and, consequently, species ecology [47]; hence, *S. wangchiachii* may possess a more complex genome than *G. przewalskii.*

Genetic diversity within species and populations is important for their sustained survival and optimal health, as it enables adaptation and modification in response to environmental changes [48,49,50]. High genome-wide heterozygosity and overall genetic diversity are strongly linked to a population’s capacity for rapid and robust environmental adaptation [49,51]. In the present study, the *P_i_* of the 10 populations of *S. wangchiachii* was low; the *PIC* (0.1818) was lower than the *PIC* (0.2562) of *S. wangchiachii* in the Jinsha River Ahai (N: 26°50′; E100°42′) and Longkaikou River sections (N27°23′; E100°31′), which have complex genetic backgrounds and mixed populations. The release of hatchery-bred fish, which often possess reduced genetic variability, into natural ecosystems may compromise the genetic diversity of native populations, thereby potentially diminishing their overall fitness and productivity [52,53]. Following the breakthrough in artificial breeding techniques in 2005, the stock enhancement and release activities of *S. wangchiachii* gradually expanded, and by the 2020s, they had become a routine large-scale ecological conservation measure in the upper Yangtze River and the Jinsha River. Based on our investigations, since 2021, the Yalong River Company Fish Stocking Station has released over 70,000 individuals of *S. wangchiachii* into these two rivers. Therefore, it may be the result of the release of captive bred individuals into the wild populations. However, the average *H_o_* (0.3244) and *H_e_* (0.2277) values of the two river populations were higher than those of *S. kozlovi* (*H_o_* 0.09578 and *H_E_* 0.06743) [29], *S. lissolabiatus* (*H_o_* = 0.2695 and *H_e_* = 0.2892) [54], *S. curvilabiatus* (*H_o_* = 0.2695 and *H_e_* = 0.2892) [55] and *S. o’connori* (*H_o_* = 0.2107 and *H_e_* = 0.1577) [56]. The above data reveal that the populations of the Yalong and Jinsha Rivers exhibit notably reduced polymorphism and genetic diversity. This pattern likely reflects a pronounced bottleneck in each population, followed by swift demographic expansion and the rapid accumulation of new mutations. However, the elapsed expansion time has been insufficient to translate these mutations into elevated nucleotide diversity. These geographic populations may have recently experienced population expansion events [57]. This phenomenon parallels the underlying cause of the limited genetic diversity observed in *Gymnocypris chilianensis* [58] and *G. przewalskii* [59].

In addition, alterations in aquatic environments and the depletion of species populations caused by overfishing and cascade development may also lead to a decrease in their genetic diversity [10,60,61]. Within freshwater systems, landscape characteristics have a more pronounced influence on the spatial distribution of genetic diversity than environmental gradients [62,63,64]. The observed negative correlation between basin-averaged slope and genetic diversity concurs with the following theory: steeper river networks can generate more volatile and less predictable hydrological regimes, which constrain the persistence of genetic variation [65]. These unstable environmental conditions can support smaller effective population sizes, consequently leading to reduced levels of genetic diversity due to increased genetic drift and reduced gene flow. Additionally, steeper river gradients act as a dispersal filter, slowing postglacial recolonization rates. This bottleneck effect, compounded by the genetic drift associated with glacial retreat, often results in diminished genetic diversity [63,66]. In our study, the BD population resided in the V-shaped canyon section, the GT population in the deep pool section, and the PZH population at the confluence of the Jinsha and Yalong Rivers. To date, more than five hydropower stations are in operation on the Jinsha River, and likewise, over five are operational on the Yalong River. The construction of hydropower stations has led to the destruction of natural habitats, thereby leading to rapid changes in the aquatic ecological environment of the region and a reduction in genetic diversity in this species. Consequently, the varied habitat conditions for each population may also influence the genetic diversity of *S. wangchiachii*.

### 4.2. Population Genetic Differentiation and Gene Flow Patterns

Geographical isolation promotes population differentiation by limiting the gene flow, a process that can be quantified using *F_st_* and cluster analyses [67]. For instance, while the habitats of *Epinephelus coioides* in the Taiwan and Guangdong populations of the striped grouper are not separated by a barrier, the approximately 600 km straight-line distance suggests the presence of geographical differentiation between the two populations [68]. On the basis of the classification standard of *F_st_* values in this study, the genetic differentiation among the 10 populations in the Jinsha River Basin and Yalong River Basin was relatively low (0 < *F_st_* < 0.05), which is similar to the results from mitochondrial *Cytb* and *Dloop* analyses [69]. However, the GT population was genetically distinct from the LZ, ML and BM populations but showed shared genetic ancestry with the DG, ZM and JC populations in our study, which may have been due to geographical isolation. Furthermore, phylogenetic analysis revealed that the populations in the upper reaches of the Jinsha River (BD, GT, SWL) exhibited significant genetic differentiation from other geographic populations, whereas the other geographic populations presented a wide range of genetic hybrid and cross-distribution patterns. This genetic differentiation pattern may be due mainly to geographical separation. The complex topography and landforms in the upper reaches of the Jinsha River (such as high mountains and valleys and rapids and dangerous shoals) may constitute an effective physical barrier, which limits the migration and gene exchange of *S. wangchiachii*, thus promoting genetic differentiation between the upper reaches and other populations. In addition, recent large-scale hydropower development in the middle and lower reaches of the Jinsha River has led to the construction of a series of cascade dams, which may further aggravate the isolation between populations [70]. The barrier effect of the dam not only directly limits the migration and diffusion of fish but also indirectly affects the genetic structure of the population by changing the hydrological regime of the river (such as flow velocity and water temperature) and habitat connectivity [55,71]. Therefore, the combined effect of geographical isolation and human disturbance may be the main reason for the significant difference between the upper reaches of the Jinsha River and the other groups.

### 4.3. Uplift of the Qinghai–Tibet Plateau and Historical Population Dynamics

Mounting evidence indicates that the uplift of the QTP has triggered extensive ecological and environmental transformations, including the onset of the Asian monsoon and the development of a temperate continental climate [72,73,74]. Our reconstruction of the demographic history of *S. wangchiachii* suggests that the shift toward a temperate and arid climate may have favored the expansion of its ancestral populations, enabling them to replace fish species adapted to warmer and more humid conditions. Subsequently, however, the effective population size (*N_e_*) appears to have declined between approximately 8 and 1 million years ago. This prolonged decline (from ~2 to 0.03 million years ago) is likely a consequence of comprehensive geological, hydrological, climatic, and ecological changes during the major phase of QTP uplift, which collectively constrained the survival and colonization of *S. wangchiachii*.

During the late stage of the second uplift (~10–4 Ma), large-scale monsoon-driven climate cycles contributed to rapid global cooling [75]. Interestingly, throughout this period, the *N_e_* of *S. wangchiachii* remained relatively stable. In contrast, the third major uplift of the QTP (~0.14 Ma) coincided with a gradual decrease in *N_e_*, and the species exhibited a pronounced demographic signal through the following three glacial cycles. The population evolutionary history of *S. wangchiachii* is highly consistent with the late stage of the Extensive Glacial Period (EGP, 0.5–0.17 Ma). The Qinghai–Tibet Plateau experienced four to five distinct glacial cycles throughout the Quaternary, including not only the EGP but also the Last Glacial Period (LGP, 0.08-0.01 Ma) and the Last Glacial Maximum (LGM, 0.021-0.017 Ma) [76,77,78]. Population expansion events of this species were primarily concentrated in the later phases of the EGP, LGP, and LGM, or during the subsequent interglacial periods. This temporal congruence suggests that Quaternary glacial–interglacial cycles were likely a key driver of population dynamics in *S. wangchiachii*. Thereafter, from the Late Pleistocene to the Holocene (~0.03 Ma), the *N_e_* of *S. wangchiachii* underwent periodic growth, during which gene flow among ancestral lineages may have been facilitated [79].

### 4.4. Adaptive Evolution and Molecular Mechanisms on the Qinghai Tibet Plateau

Whole-genome duplication exerted a pervasive evolutionary influence, furnishing a vast reservoir of raw genomic material for the genesis of evolutionary novelty and catalysis of subsequent diversification [80]. The genome evolution of *Gymnodiptychus pachycheilus* has accelerated, and genes whose lineage has undergone rapid evolution and positive selection characteristics are enriched in functions related to energy metabolism [81]. Relative to a lowland fish such as *Ctenopharyngodon idellus*, all examined Schizothoracine fishes demonstrated a significant increase in dN/dS ratios. Notably, the evolutionary rates of specific Gene Ontology terms associated with altitude adaptation—including energy metabolism, hypoxia response, and DNA repair—were significantly accelerated [82]. These molecular patterns indicate that fish inhabiting the Qinghai–Tibet Plateau have undergone rapid adaptive evolution. In the present study, *S. wangchiachii* exhibited unique adaptability to the unique environment of the plateau, and water temperature was among the most critical factors. Water temperature is closely related to the metabolic intensity of fish and is an important environmental factor affecting their physiology, biochemistry, and growth and development [83]. Several molecular pathways have been implicated in the high-altitude adaptation of fish. Specific genes under selection are involved in regulating responses to temperature variation through mechanisms such as signal transduction, energy metabolism, and membrane activity [73,84]. Specifically, transcriptome research on *G. namensis* [85] and *G. selincuoensis* [86] has yielded similar results. Our analysis suggests that the large-scale uplift of the Qinghai–Tibet Plateau may have given *S. wangchiachii* an advantage in adapting to environmental changes [72].

In our study, the populations of *S. wangchiachii* with common molecular pathways between selected SNPs were enriched in metabolic GO terms (such as sodium ion transport and cellular response to inorganic substances) in Group 1 vs. Group 2, Group 1 vs. Group 3, and Group 2 vs. Group 3. The temperature-related SNPs revealed were involved mainly in gene functions, such as mitochondrial translation and transport and energy metabolism. Temperature may cause various physiological responses in *S. wangchiachii*, resulting in highly active gene transcription. High mitochondrial gene concentrations have also been studied in other warm-adapted animals. Compared with Group 3, Group 2 was enriched in more metabolism-related GO terms, such as N-acetylneuraminate metabolic process corticospinal neuron axon guidance. Additionally, variation in the expression of gene pairs could contribute to enhanced organismal fitness and rapid adaptation to novel selective pressures. Consequently, evolutionary forces have likely sculpted the tetraploid genome of *S. wangchiachii* by selectively retaining duplicated genes that confer environmental resilience. Decoding this genome will therefore deepen our understanding of how polyploid fish genomes evolve in response to shifting ecological conditions.

## 5. Conclusions

In the present study, we analyzed the structure and genetic variation of *S. wangchiachii* using GBS technology. The genetic diversity of each population was relatively low based on the analysis of the selected SNPs. The genetic differentiation among the 10 populations was not significant, but cluster analysis revealed that the populations from the Jinsha River Basin and Yalong River Basin showed significant clustering, indicating that geographical distance and hydropower development have affected genetic differentiation among the populations. The analysis of historical population dynamics showed that with the uplift of the Qinghai–Tibet Plateau, *N_e_* of *S. wangchiachii* first increased but then decreased. In addition, the enrichment of selected sites and energy metabolism and cellular processes contributes to adaptation to high-altitude low-temperature environments. The results of this study provide a data for research on the resource conservation and adaptability of *S. wangchiachii* in the upper reaches of the Yangtze River.

Protecting and maintaining the high genetic diversity of *S. wangchiachii* in wild populations may be critical for the future. First, the upstream of Jinsha River (including BD, GT, and SWL populations) are likely to be an important refuge for *S. wangchiachii* and should be established as a nature reserve. Then, in populations with extremely low genetic diversity (for example, SWL and SG), hatchery enhancement programs based on genetic management should prioritize the use of broodstock sourced from the same river basin. Furthermore, the relevant fisheries authorities should conduct ecological assessments of small dams and diversion-type hydropower stations on the Jinsha River and Yalong River, prioritizing their removal or the installation of fish passage facilities to restore historical gene flow corridors. Last, we suggest that priority should be given to protecting natural river reaches at the confluences of different water systems, in order to maintain potential opportunities for gene flow and prevent further genetic differentiation caused by increased habitat fragmentation.

In addition, the present study has a small sample size and thus limits the genetic information. In future study, it is necessary to expand the sample size to further screen for more positive SNPs.

## Figures and Tables

**Table 1:** Specimen information.

Tributary	Sampling Site	Abbreviation	Coordinates	Altitude (m)	Number of Samples
Jinsha River	Benda, Sichuan	BD	N: 32°39′0.13″ E: 97°30′21.01″	3408	10
	Gangtuo, Sichuan	GT	N: 30° 0′10.57″ E: 99° 6′20.45″	3316	7
	Suwalong, Sichuan	SWL	N: 29°24′01.5199″ E: 99°03′48.1586″	2395	4
	Shigu, Sichuan	SG	N: 26°53′01.40″ E: 99°57′35.8″	1818	10
	Panzhihua, Sichuan	PZH	N: 26°52′23.01″ E: 101°48′6.80″	983	10
	Wudongde, Sichuan	WDD	N: 26°15′49.6″ E: 102°44′0.74″	795	6
Xianshui River	Xianshuihe, Sichuan	XSH	N: 30°12′6.33″ E: 101°1′50.8″	2685	6
Yalong River	Yajiang, Sichuan	YJ	N: 30° 1′56.39″ E: 101°0′52.32″	2578	5
	Ganzi, Sichuan	GZ	N: 31°37′10.1074″ E: 99°54′26.9254″	3360	8
	Xinlong, Sichuan	XL	N: 30°56′24.02″ E: 100°18′41.89″	3276	9

Notes: N, north; E, east.

**Table 2:** Genetic diversity values among *Schizothorax wangchiachii* populations.

	Population	*H_e_*	*H_o_*	*Pi*	*PIC*
Jinsha River	BD	0.2336	0.3281	0.2473	0.1868
	GT	0.2306	0.3408	0.2495	0.1833
	SWL	0.2200	0.3350	0.2538	0.1738
	SG	0.2206	0.2632	0.2355	0.1784
	PZH	0.2365	0.3387	0.2496	0.1894
	WDD	0.2216	0.3173	0.2440	0.1766
Yalong River	XSH	0.2312	0.3411	0.2538	0.1838
	YJ	0.2268	0.3325	0.2539	0.1804
	GZ	0.2268	0.3231	0.2442	0.1814
	XL	0.2293	0.3243	0.2445	0.1836
Average		0.2277	0.3244	0.2476	0.1818

Notes: *H_e_*, expected heterozygosity; *H_o_*, observed heterozygosity; *P_i_*, nucleotide diversity; *PIC*, polymorphism information content. GT, Gangtuo; SWL, Suwalong; SG, Shigu; PZH, Panzhihua; XL, Xinlong; GZ, Ganzi; XSH, Xianshuihe; YJ, Yajiang; WDD, Wudongde.

**Table 3:** Genetic distance (above diagonal) within and between *Schizothorax wangchiachii* populations and the genetic fixation index (*F_st_*, below diagonal) of the populations.

Population	BD	GT	SWL	SG	PZH	WDD	XSH	YJ	GZ	XL
BD	-	0.0023	0.0108	0.0256	0.0384	0.0502	0.0393	0.0382	0.0379	0.0398
GT	0.0023	-	0.0084	0.0396	0.0349	0.0463	0.0388	0.0383	0.0522	0.0540
SWL	0.0108	0.0083	-	0.0394	0.0286	0.0369	0.0399	0.0376	0.0084	0.0567
SG	0.0252	0.0388	0.0386	-	0.0488	0.0599	0.0421	0.0394	0.0190	0.0213
PZH	0.0377	0.0343	0.0282	0.0477	-	0.0112	0.0285	0.0228	0.0457	0.0462
WDD	0.0490	0.0452	0.0363	0.0582	0.0112	-	0.0400	0.0308	0.0591	0.0590
XSH	0.0385	0.0381	0.0391	0.0412	0.0281	0.0392	-	0.0173	0.0255	0.0261
YJ	0.0375	0.0376	0.0369	0.0386	0.0225	0.0303	0.0171	-	0.0352	0.0359
GZ	0.0372	0.0509	0.0546	0.0188	0.0447	0.0573	0.0252	0.0346	-	0.0006
XL	0.0390	0.0526	0.0551	0.0211	0.0451	0.0270	0.0257	0.0353	0.0012	-

Notes: BD, Benda; GT, Gangtuo; SWL, Suwalong; SG, Shigu; PZH, Panzhihua; XL, Xinlong; GZ, Ganzi; XSH, Xianshuihe; YJ, Yajiang; WDD, Wudongde. The lower triangle represents the coefficient of genetic differentiation between populations (*F_st_*), and the upper triangle denotes the genetic distance between populations (*D*).

## Data Availability

The data presented in this study are available on request from the corresponding author without any restrictions.
